# Lives Penciled in, the Reality of Chronic Health Conditions and Trauma: Reflexivity, Health, and Shadowed Identities

**DOI:** 10.3389/fpain.2022.903724

**Published:** 2022-06-22

**Authors:** Richard Bruce Hovey, Veeresh Pavate, Marie Vigouroux

**Affiliations:** ^1^Faculty of Dental Medicine and Oral Health Sciences, McGill University, Montreal, Quebec, QC, Canada; ^2^Department of Pediatric Anesthesia, Edwards Family Interdisciplinary Centre for Complex Pain, Montreal Children's Hospital, Montreal, QC, Canada

**Keywords:** chronic pain, cancer, thalassemia, posttraumatic stress disorder (PTSD), hermeneutics, metaphors

## Abstract

When living with chronic health conditions or experiences of trauma our lives can become perpetually penciled in. The use of the penciled-in metaphor means to arrange our time tentatively: a date, an appointment, a meeting, seeing a movie, or attending a class. In our technologically-driven world of electronic calendars where everything is entered electronically, the utility of the pencil and hand-written agendas have all but vanished. However, for the purpose of this article, the pencil provides a metaphoric common ground to learn about the totality of the disruption experienced by living with chronic health conditions and their residual trauma. The pencil is touchable, tangible and as a researcher and a person who lives with challenging health concerns, metaphors help me to create an understanding of the chaos of living a life in pain with cancer. This article is a person-centered account of the process of reflexive coping and self-processing of pain by a pain researcher and educator. This article focuses on the metaphor of penciled-in lives to provide a qualitative account of experiences of pain from chronic health issues and the trauma both physical and emotional it causes. This act of reflexivity becomes a personal examination of life. It reveals to me my beliefs, decisions, and practices before and during my hermeneutic journey and how these may have prejudiced my thinking and behaviors.

## Enigmatic Relationship With Health

As a person living with chronic pain and cancer, I learned that health resists universal definition because it can only be interpreted, constituted, and reconstituted through specific professional, personal, promotional, educational, cultural, governmental, and communal lenses. The components of health are complex, with multiple assessments and dimensions. For example, I can identify the usual suspects of health such as physical, occupational, spiritual, emotional, intellectual, social, sexual, mental health, and of course aging. However, during various stages of our life, we develop a sensibility (the ability of sense) of knowing when one's health is waning. We might say that we are not feeling right today, something is off; “I feel unwell.” This awareness is based on how we feel when compared to how we typically navigate our worlds with anticipated expectations. Similarly, the expression of pain physically varies among people simply because they experience their health differently. When we attempt to diagnose illness, it is done as what is expected, or of concern based on biomedical test results. Therefore, although the possibility to diagnose with accuracy is a powerful resource of modern medicine. It is what saves many people with early detection. Living with pain and cancer means establishing a new self-determined sensibility of what personal health feels like. We still need to establish when our health shifts to illness, just differently (1).

There are challenges implicit in defining personal lived health precisely and adequately because health is an overarching conceptualization inherent within the human experience (2, 3). Health remains irreducible and resistant to the certainty of a purely positivistic objective definition (4–6). Health resides within us as reflective human beings. Nevertheless, this does not negate the value of discipline-specific definitions that provide direction, focus, and professional enculturation for healthcare practitioners, health promoters, and educators.

This metaphoric explication is of particular interest for people living with chronic pain and cancer where a recalibration of homeostasis is required. Since the new way of experiencing life means having a level of pain and uncertainty. The penciled-in metaphor offers a reflection on how pain and cancer have affected my life. This is just as they were as unique people before pain and cancer change my life. The way each person pencils in their life events, erases many, wears away the time of their life faded away helped by the tears that fall to view a shadowy reminder of oneself (7).

### Metaphors as a Window Into Understanding

The word metaphor is a figure of speech by which a characteristic of one thing or topic is allocated to another. Greek word *meta* means over and across and *pherin*, to carry or bear. Together the Greek word *metapherin* means to transfer, carry over, change, alter, or use a word in an unusual way. This transfer of meaning is different but approximating it or comparable to help create an understanding of another topic or experience. The metaphor helps to consider that which is similar and that which is different while transferring meaning. In qualitative research, the metaphor becomes a technique to explore complex and sensitive human experiences such that they might become more apparent to the reader. The reader may not have had that experience and so finding a metaphoric common ground adds to the art of understanding at the heart of philosophical hermeneutics. For others who have had a similar experience such as loss and pain, it may help to validate their experiences and help them deepen their reflection on that with which they live (8).

Ricoeur writes, “If we can incorporate the surplus of meaning of metaphors into the domain of semantics, then we will be able to give the theory of verbal signification its greatest possible extension” (9). In other words, the metaphor invites reflection and can become a bridge to extend our understanding of something where words are not readily available to explain or interpret life challenges. The life penciled-in metaphor is my personal reflexivity moving from my internal thinking to being available to others for reflection and interpretation.

The metaphor provided below was adopted as a means to poetically illustrate the often unknown aspects of living with chronic pain, that of loss of choice, exhaustion, anxiety, depression, and trauma.

### Reflective Metaphors

When I recall attending school as a much younger person, I remember having several freshly sharpened pencils readied for action on my desk in preparation for class to begin to take notes, doodle, and dream. I especially used them for drafting documents to be inked in or typed out later. I was not seeking pencil perfection. In a precomputerized world, the pencil provided a means to draft-write, draw, create, and offer thoughts with the option to erase and re-write over the old text. Our papers became shadowy imprints of what was written on the page, erased, and then written over, as new ideas toppled old, while there remained the remnants of the past writing, like a fading memory. The use of this metaphor is explicitly chosen to state that when I transitioned from acute to chronic pain, with a subsequent diagnosis of advanced metastasized prostate cancer, everything in my life changed, some quickly, others slowly. Much like the fragility of the act of erasing pencil marks from paper, there is the reflection of erasing my life expectations that take place. Living with chronic pain and cancer, I literately feel worn out, erased, folded, and creased as does the paper one works over and over, becoming exhausted and more fragile. My life as the written page metaphorically shrinks further and further into the shadows of my previous self, unless, beyond recovery; a healing narrative is all that is left (10). The fragility of health and identity become charcoaled and blurred.

### The Fragility of Pencils

The lead within the pencil keeps the clarity of presentation through the applied tension and skillful pressure of the sharpened point onto the paper. Too much pressure on the pencil translates into a possible breaking point of the lead and tearing through the paper. As a person living with challenging health conditions, the transition from prepain to a pained life with cancer demanded changes. These changes were not by choice but provoked by the sheer desire to live well and find a new equilibrium and life. Too much stress and pressure from both internal (my sense of self) and external sources (demands of work and society) can disrupt this new sense of acquired equilibrium, sometimes to a breaking point of self, leading to a rupture through the paper. Without healing, when a cure is not possible, each metaphorical sharpening of the pencil shortens it and in a similar way, we eventually are left short of living a meaningful life. If the pain and cancer cannot be disrupted or arrested, we remain confined by our pain (11). “We notice how pain and the suffering it inflicts change in character when they are no longer accompanied by the certainty or the expectation that it can be eliminated” (4). The penciled-in metaphor illustrates that even though life is not always predictable or certain, we still can create possibilities through our choices as an altered sense of security even predictability. Much of this becomes erased and tattered over time while our pain dictates many of our losses and lack of choice.

One of my graduate students, Mr. Veeresh Pavate offers the following reflection about living with Thalassemia, not necessarily only physical pain but the pain of time and treatments.

My agenda works on a 3-week schedule, and it is always penciled in advance. Being a person with thalassemia, a chronic inherited genetic health condition, I require timely blood transfusions. This allows me to accomplish my personal and my professional goals. As my body does not produce enough healthy red blood cells, it needs external assistance which means I have always led my life on a borrowed timeline. It entails the need for two units of blood transfusion every 3 weeks. This process takes 4–5 h on an outpatient basis at the hospital. Hence, as we keep writing with a pencil lead which not only becomes smaller with usage, the pencil needs to be re-sharpened to extend its life. That is the same feeling I feel every third week as I get tired and it becomes challenging to concentrate on my research work, the penciled-in date in my agenda approaching for my transfusion. After this date, I get the energy to continue on high octane for a couple of weeks and go through the 3-week cycle once again. This sort of hard-etched script is sometimes challenging for others in society to understand. The complex experience of having a chronic health condition and the trauma resulting from it is not easy for society to appreciate. In fact, my observation and life experiences have been that society has a preconceived (or already penciled in) image of people living with chronic health conditions: they cannot have the very same aspirations and ambitions as people with reasonably good health. When one experiences such things in life, one wishes that it was as easy as turning the pencil around to use the eraser to scrub away the experiences one has gone through. It is always a struggle to make others see us as people.

### Pain and Cancer Experiences as Reflective Expressions

Every person living with chronic pain knows something of the profound inwardization involved in suffering and the endurance of pain (6). When we humanize our approach to qualitatively researching chronic pain, we carefully and sensitively begin to gain insight and understanding into another person's world with its substantial and oppressive magnitude of pain's effect. The complexity of life changes suddenly, profoundly with its effect on the whole person with its multidimensionality, “dragging us downward toward those dark demons, which our medical colleagues may if unable to find success with chronic pain describe in terms of hypochondria and depression” (4). If we listen carefully to others, we begin to learn how that chronic pain and cancer and the trauma they inflict, change the person's perspective of health (12, 13).

Continuing with the life penciled-in metaphor, one could say that, before living with chronic pain, the person used to pencil in only a few things at any given time, identifying them as uncertain. Life, for the most part, was lived with some certainty; where classes were scheduled, working hours printed out, going out to movies with friends, dinner with family, a next vacation destination, going for a walk, and feeling mostly well. This generalization illustrates the transition from a life with minor to then major disruptions; an existence of isolated pain and suffering to one where there are no longer only occurrences. Pain and cancer are now pervasive and persistent. As described by Ganadmer: “So powerfully does pain cause us to withdraw from all external experience of the world and turn us back upon ourselves” (4). We lose our sense of belonging in our own world and suffer in so many ways (14). It begins with physical pain but stretches perversely into all aspects of life. I am myself penciled into my own life. I ask myself: who am I now and who will I become?

Chronic pain ruthlessly changes a person's life. The intensity, duration, and perception of the pain dictate the degree of life that is lost, unlived, and forgotten. We fade away from who we thought we were, before chronic pain. The things that mattered to us begin to slip away over time with the pain. Jobs, school, friends, and all the other activities that we previously enjoyed without thought or concern now challenge us in ways never preconceived. Even if the pain is controlled over time, the loss of opportunity, personal goals, and a projected future of family and friends may change, fade away, or at best may be re-negotiated and perhaps partially returned (15).

### Time Reflections

Researching chronic pain means researching the whole person, qualitatively and quantitatively. The whole person is complex and as such, the term chronic entails the time spent with the illness, expressed in months, years or decades. It refers to chronological or sequential time. To deepen the penciled-in metaphor, we offer a reflection on changing perceptions of time when dealing with chronic illness and its resulting trauma.

Chronos, one of the Greeks' conceptualizations of time, is the time when we engage directly with a structured time and place. It can be linked to the medical world, as primarily a quantitative concept (16–18). When we make an appointment with a medical expert, a counselor, or a physical therapist, for example, we arrive at a prearranged time, have medical tests, hear about diagnosis, discuss continuing or new treatment options, and then leave at the end of the appointment. Chronos is about preciseness, such as the exact dose of medicine, combinations, and our body's response. It is the marked time between medical visits, the time between taking medications and waiting for the effects and the unfortunate side effects, and the time in between finding pain and forgetting about it. These are moments in a time dominated by the promise of medicine, cure, and recovery where one anticipates the eradication of pain.

Regarding healthcare and its help with reducing the pain of the pained person Gadamer writes,

This is something we know from contemporary medicine with its virtuosic capacity to “eliminate” pain, the source of the pain, the symptom, and sometimes even more than this. By means of its capacity to remove pain in this way modern medicine changed the role and importance within human life of certain illnesses, which can be so quickly dealt with today. One simply takes something for it and then it is gone (4).

Advancing the pencil metaphor, Chronos demands to pinpoint accuracy. The pencil needs to be at its sharpest to ensure that medical appointments are precisely scheduled, medications are accurately measured, with periods and commas not to be confused. For the person living with pain, being at their sharpest is challenging but necessary within Chronos time. But at what cost? Remembering the small pencil sharpeners, the continuous sharpening of the pencil which shortened the pencil each time, leaving an ever-present pile of shavings. This metaphor is transferable because too much of Chronos time for the person living with chronic pain translates into fatigue from being driven to meet expectations of self and others, a gradual sharpening. Regrettably, ending up put in a heap of our own, exhausted and possibly demoralized by the demands of Chronos. I have ended up in this metaphor pile there many times over the last decade while trying to keep up with the expectations dictated by my old life (10).

Kairos can be thought of as the length of time lived in-between Chronos time, which is far more extensive, where days, weeks, months, years, and tears can pass by without relief from our pain. The metaphoric pencil lead is allowed to dull here with wider marks on our paper of life. It eases the tension on the person and provides the time to reflect and create new passageways for life. This time becomes an experience in which there is the possibility of creating new meanings, ways of living, and healing of our souls. We experience time as Kairos, through the qualitative or hermeneutic time. For the person living with chronic pain, that experience is one of living in-between, which is the locus of hermeneutics, medical visits, treatments, and a life penciled in all the while punctuated by Chronos. For a more in-depth understanding of hermeneutics, please see the following references (4, 5, 15). Kairos time is where people living with chronic pain spend most of their lives; waiting, wondering, and hoping, while exhausted by the pain and trying to make sense or meaning from their experience. This unfolds while the unbridled Chronos is ticking away our lives. Returning to the pencil metaphor, this can be compared to the in-between: the point where the pencil is no longer sharp enough for Chronos but has much writing potential still left in the lead. This in-between time is where Kairos expands its potential into the creative possibility of writing poetry, prose, journaling, artistic doodling, sketching, or just dreaming of improving one's life. The sharpening of the pencil becomes a necessary annoyance that may disrupt the creative process, but we are still able to fall back into this hermeneutic experience. When a person cannot be cured, we can still help to heal them. Kairos is also where non-medicalized interactions may occur: pain support groups, alternative therapies, socializing within group activities, and perhaps even finding temporary peace. When not focused on a perpetually sharpened pencil, Kairos uses the whole of that lead until it really needs to be sharpened thereby extending the longevity of the pencil and, in other words, humanizing the realities of life for the person even if only for a short time.

### Chronos vs. Kairos

The brevity of the healthcare encounter favors Chronos over Kairos. For example, take the Likert Scale to help assess pain. Each number from Chronos represents a perceived level of pain experienced by the person. However, it becomes obvious that a number only represents a minimalistic representation of the experience of health. The number from these scales can be thought of as a dam holding back the lived experiences of that person. Behind the number, the ever-increasing levels of water represent how all the dimensions of health are interwoven and affecting the whole person now. When I recall my transition from acute to chronic pain, the Likert Scale seemed to make more sense as I could recall being relatively pain-free. However, after close to a decade of chronic pain, the scale becomes less effective as a means of expressing my pain. My pain narrative is a much better way to express it. This kind of narrative needs practice, such that the patient and clinician become interpretative partners to cocreate a shared understanding of not only the perceived level of the pain, but what other dimensions of their health are influencing their health experiences. This co-creation can be facilitated by the use of metaphor.

When a person living with chronic pain offers a numeric answer from 1 to 10 out of 10, each Likert scale number (Chronos) is covering up Kairos, the collective manifestation of how all our dimensions of health are influencing our pain. As we move up the Likert scale, the energy to hold life together can become overwhelming. Time experienced in excessive pain cautions healthcare that the worse possible outcome is possible: suicide. The treatment of one health issue creates others as undesirable side effects: the lesser of two health-related outcomes. Treating the narrative beyond the chosen number becomes whole-person care. If a person living with pain can assemble a diverse community of healthcare providers who can help with each dimension of health, then one might find a new sense of wellbeing and life again.

Another of my graduate students, Ms. Marie Vigouroux explains her relationship with time and painful trauma.

Somewhere along the line of living trauma and getting diagnosed with posttraumatic stress disorder, my relationship with time shifted greatly. I spent most days consciously and unconsciously avoiding situations that could cause more trauma, anxiously waiting for more trauma to happen. I put aside my big life plans, only letting life happen to me instead of actively taking part in creating the life I wanted for myself. I lived almost exclusively in Chronos, constantly anchoring myself to sharply written events in my agenda, unable to spend and invest time on my own healing. From an onlooker's perspective, I was functional: I held down a job, I maintained relationships, I cared for my pets. It is only when healthcare workers began interesting themselves in my experience of the time in-between that it became apparent to them that I had been living with posttraumatic stress.

Although my life remains still penciled in and tentatively lived, as the pandemic continues into 2022, we are left with the work of finding joy in life. This current article does not explore pain during a pandemic as it appears in other journal articles, but rather acknowledges pain as it manifests in many ways for many people.

### We Cannot Overstep Our Own Shadow and Colored Pencils

Moving our thinking from the dullness of the charcoal pencil to colored pencils metaphorically offers people hope of exploring new and brighter options for their lives. A word of caution is that regardless as we explore time, it passes, and we get older with the disabling effects that are somewhat inevitable. The experience of pain in an aging body to me means not waiting until the pain is better, but learning to live it. Gadamer explains:

An awareness of time—this is something momentous. For it does not signify merely an increase of knowledge, in the power of anticipation, but involves what is in fact a fundamentally different status altogether. It means the ability to forgo the gratification of the most immediate goal in favor of a long-term fixed purpose (4).

This means to keep seeking out new and different ways to experience the world with a fresh perspective, using new paper and colored pencils with which to re-story ourselves (19, 20). Remember: we cannot overstep our shadows because we always are reflectively everything that we are. Reflexivity reminds us to confront ourselves and our understanding of our life circumstances. This is a lifelong process that ebbs and flows, and we as humans are inherently reflective beings. A constant renewal becomes possible; who we were, who are, and who we can become with colored pencils to narrate a new self, coloring outside the lines means exploring new and meaningful life possibilities (21–23). Perhaps coloring outside of the metaphorical lines of our lives will mean something different to everyone; this is where its potential exists. The only thing I can be certain of is that my pain will be there with me as I continue as a work in progress.

## Conclusion

The intention of the authors in writing this manuscript was to make the experience of chronic illness accessible to those researchers and clinicians who may never have experienced it. By hermeneutically exploring this experience through the penciled-in metaphor, the authors hope that people who have never experienced chronic illness will be able to better understand it. Humanizing the experience of chronic illness is necessary for its scholarship, and a provides a foundation upon which researchers can produce patient-centered research, and clinicians can provide patient-centered care.

## Data Availability Statement

The raw data supporting the conclusions of this article will be made available by the authors, without undue reservation.

## Ethics Statement

This study was reviewed and approved by the Institutional Review Board of McGill University. Written informed consent was obtained from all participants for their participation in this study.

## Author Contributions

RH conceptualized this manuscript. All authors contributed to the drafting and editing of the manuscript.

## Funding

The authors wish to acknowledge the financial support of the Quebec Pain Research Network (QPRN) for the publication of this article.

## Conflict of Interest

The authors declare that the research was conducted in the absence of any commercial or financial relationships that could be construed as a potential conflict of interest.

## Publisher's Note

All claims expressed in this article are solely those of the authors and do not necessarily represent those of their affiliated organizations, or those of the publisher, the editors and the reviewers. Any product that may be evaluated in this article, or claim that may be made by its manufacturer, is not guaranteed or endorsed by the publisher.
